# Supplementary material for: A kinetic method for measuring agonist efficacy and ligand bias using high resolution biosensors and a kinetic data analysis framework
Source: Sci Rep. 2020 Feb 4;10:1766. doi: 10.1038/s41598-020-58421-9 (PMC7000712; doi:10.1038/s41598-020-58421-9)
Supplement: Supplementary file 1 — Supplementary Information. [file 41598_2020_58421_MOESM1_ESM.pdf]

Supplementary Information

A kinetic method for measuring agonist efficacy and ligand bias using high resolution biosensors and a kinetic data analysis framework

Sam R.J. Hoare[1], Paul H. Tewson[2], Anne Marie Quinn[2] and Thomas E. Hughes[2]

[1] Pharmechanics LLC

14 Sunnyside Drive South, Owego, NY 13827 USA

[2] Montana Molecular

366 Gallatin Park Dr. Suite A, Bozeman, MT 59715 USA

Supplementary Table S1. Arrestin recruitment association exponential fit parameter values for the AT_1 receptor. Time course data for arrestin recruitment stimulated by the test ligands at 32 μM concentration (see Fig. 2 and Supplementary Fig. S6) was fit to the association exponential equation $y = \text{Plateau} \times (1 - e^{-k_{obs} \cdot t})$ to determine Plateau and k_{obs}. [1]NFU, normalized response units.

Ligand	Plateau (NFU [1])	k_{obs} (min^{-1})	$t_{1/2}$ (sec)
AngII	0.43 ± 0.01	0.94 ± 0.07	45 ± 3
TRV055	0.44 ± 0.00	0.85 ± 0.05	49 ± 3
TRV045	0.42 ± 0.01	0.90 ± 0.14	49 ± 9
TRV026	0.40 ± 0.01	0.62 ± 0.08	69 ± 8
SII	0.38 ± 0.02	0.53 ± 0.13	84 ± 21

Supplementary Table S2. Concentration response analysis of arrestin recruitment at single time points and for the initial rate of recruitment. The arrestin sensor response to various AT_1 receptor ligands was measured at single time points of 1 minute (representing the rise phase) and 20 minutes (representing the plateau) after addition of ligand, as described in Fig. 4. Data were fit to a sigmoid curve equation to determine E_{max} and EC_{50}. These data are compared here with the kinetic model fit to the concentration-response of the initial rate (right-hand two columns, from Table 1).

	1 minute time point		20 minute time point		Kinetic model initial rate	
Ligand	E_{max} (% AngII)	EC_{50} (nM)	E_{max} (% AngII)	EC_{50} (nM)	Emax (% AngII)	K_A (nM)
AngII	100	110	100	17	100	120
TRV055	103	100	100	14	93	130
TRV045	101	200	100	25	89	300
TRV026	84	150	99	23	62	180
SII	64	620	88	210	48	1,100

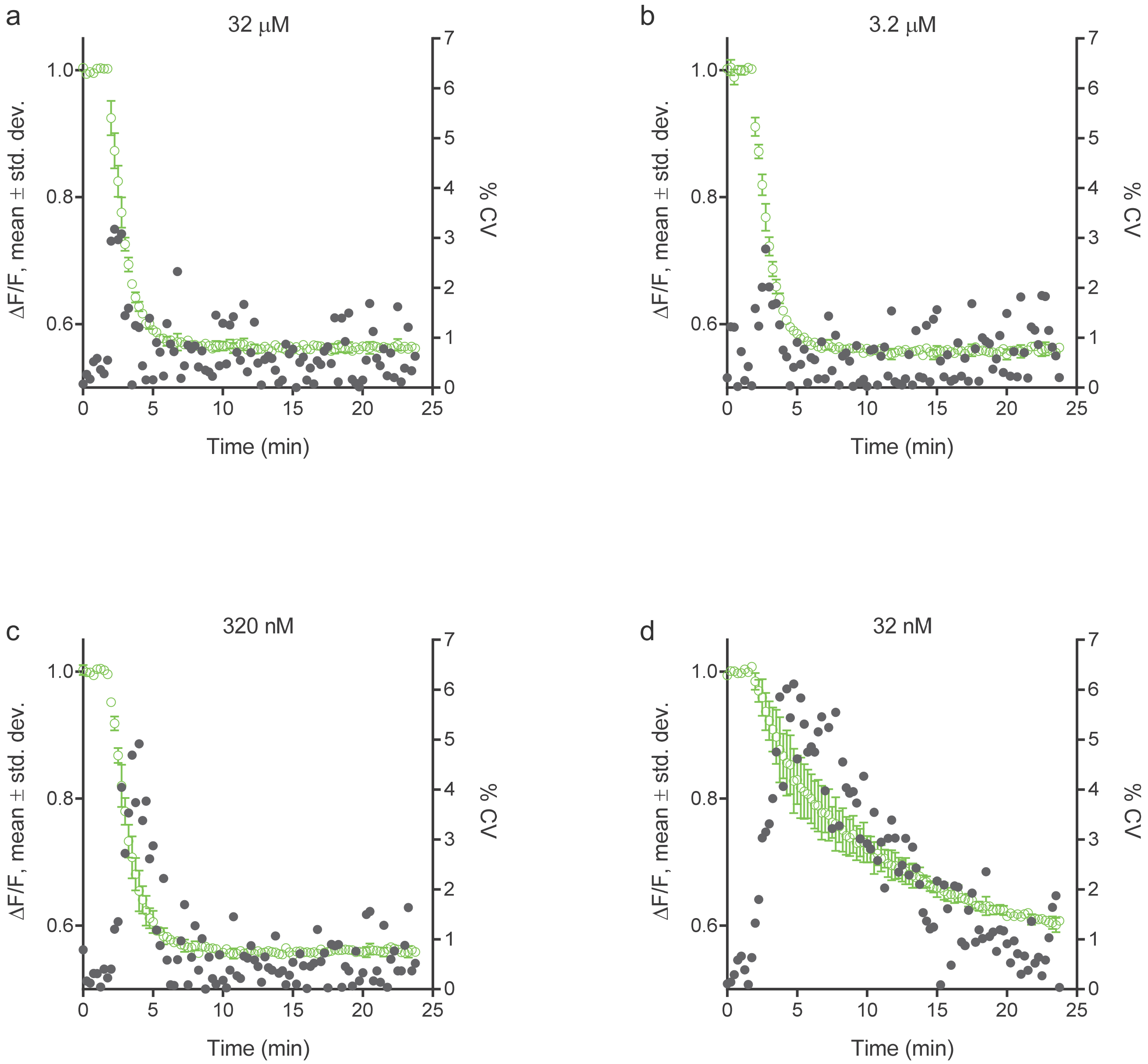

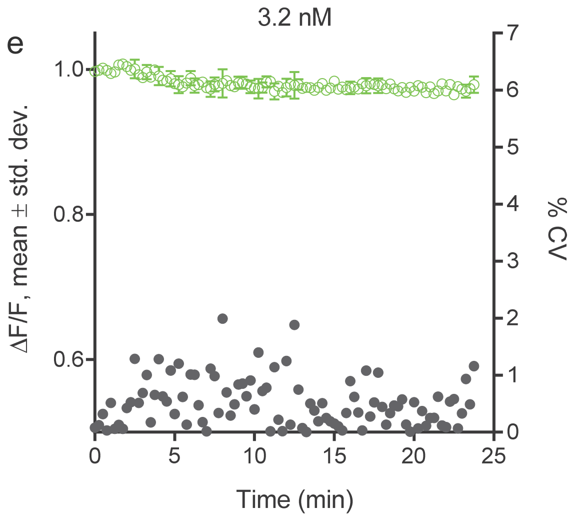

Supplementary Figure S1. Characterization of within-experiment variability between technical replicates, arrestin sensor stimulation by 10 μM AngII via the AT_1 receptor. Data were normalized to baseline (ΔF/F) as described in Fig. 1. Data points are duplicates, with error bars representing the standard deviation. The % CV was calculated for each data point and is plotted on the right-hand y axis. The analysis was performed for multiple concentrations of AngII, spanning the effective range (a, 32 μM; b, 3.2 μM; c, 320 nM; d, 32 nM; e, 3.2 nM). The time interval between data points was 15 seconds. Fluorescence intensity was measured using the BMG CARIOstar and data normalized to baseline fluorescence before agonist addition.

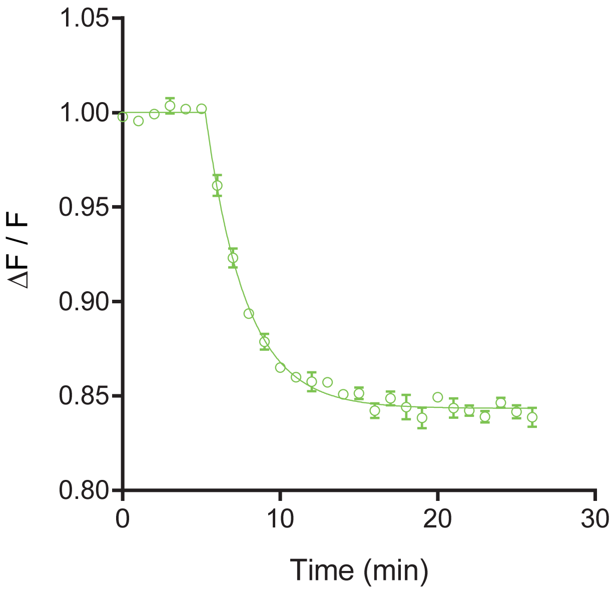

Supplementary Figure S2. Arrestin sensor response via the β_2-adrenoceptor in response to 10 μM isoproterenol. Cells were transduced with viruses encoding the receptor, the sensor and GPCR kinase-2. Data were generated with the Biotek synergy Mx plate reader. Data points are mean ± sem (n = 4). The signal was normalized to baseline; specifically it was quantified as the fluorescence after agonist addition divided by that of the baseline signal before addition (ΔF / F).

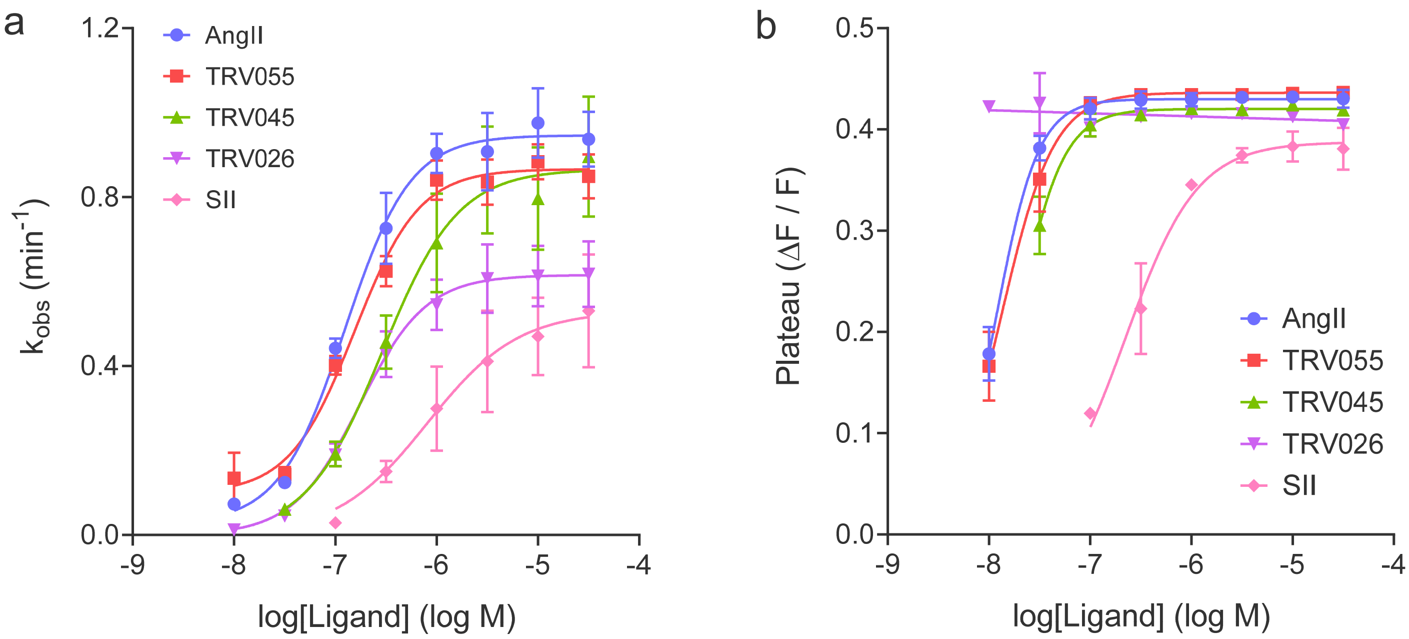

Supplementary Figure S3. Curve fit parameters for the time course of arrestin recruitment to the AT_1 angiotensin receptor by various ligands: (a) k_{obs}, (b) Plateau. The time course data (Fig. 2a and b, Supplementary Fig. S7) were fit to the association exponential equation:

$$y = \text{Plateau} \times \left(1 - e^{-k_{obs}.t}\right)$$

The measured values of the observed rate constant k_{obs} and Plateau are plotted against the ligand concentration. Note the value of both parameters increases as the ligand concentration increases. The curves are fits to a sigmoid dose-response equation.

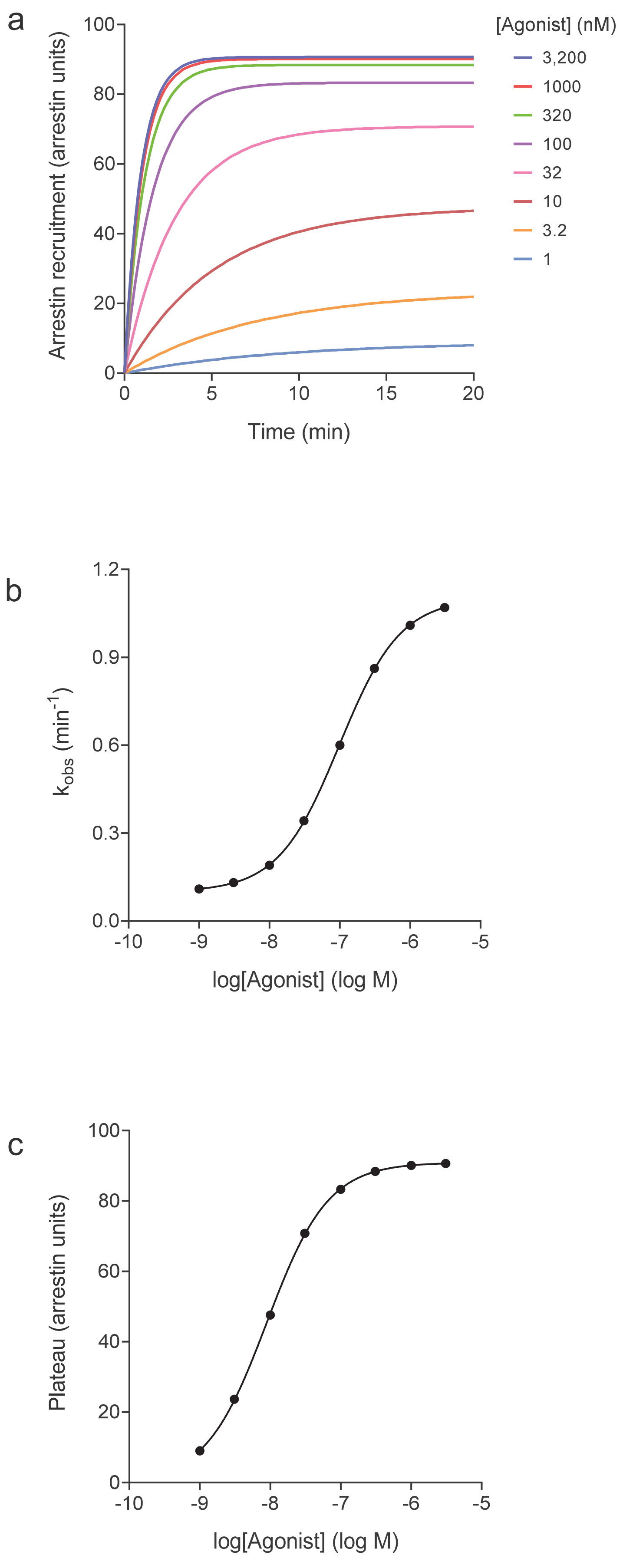

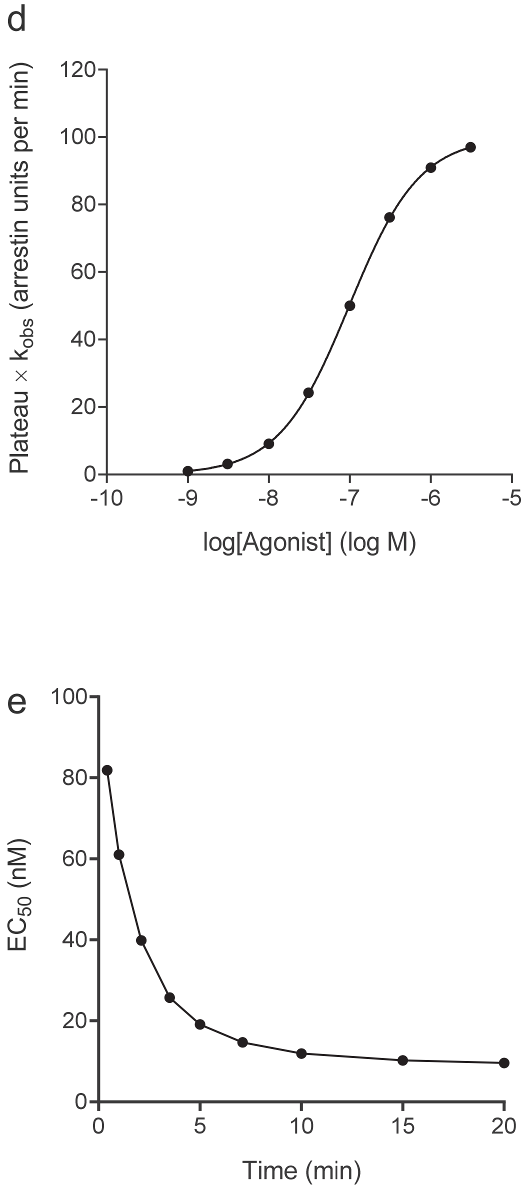

Supplementary Figure S4. Simulated arrestin recruitment data. The arrestin recruitment model (Appendix) was used to simulate the time course of arrestin recruitment to a GPCR stimulated by agonist ligand. Time course data (a) were simulated using equation (1), assuming receptor is in excess over arrestin, for a range of concentrations of agonist. From these data the observed rate constant k_{obs} and Plateau can be calculated using equation (3). The calculated values are plotted versus the agonist concentration (b, k_{obs}; c, Plateau). Note the value of both parameters increases as the ligand concentration increases. The curves are fits to a sigmoid dose-response equation. The initial rate of recruitment at each concentration can be determined by multiplying the Plateau by k_{obs}. The resulting value is plotted against the agonist concentration (d). The data are then fit by the sigmoid dose-response equation to determine k_τ (k_{obs} × Plateau at maximally-effective [A]) and agonist affinity for the receptor ($[A]_{50}$). (e) Change of EC_{50} over time. Arrestin response versus agonist concentration data were fit to a sigmoid dose-response equation to determine EC_{50} at the indicated times. Model parameter values used for the simulation were: K_A, 100 nM; $[R]_{TOT}$, 100 receptor units; $[N]_{TOT}$, 100 arrestin units; k_N, 0.01 receptor units^{-1}min^{-1}; k_{-N}, 0.1 min^{-1}. The calculated k_τ value was 100 arrestin units.min^{-1}.

Supplementary Figure S5 Quantifying k_τ concentration response method

➊ Measure arrestin recruitment over time at multiple ligand concentrations

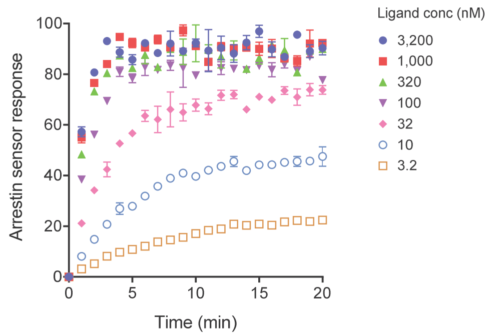

➋ Fit data to association exponential equation

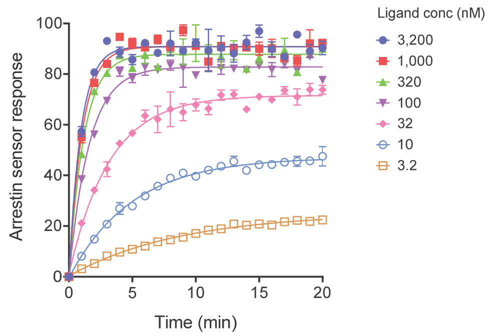

➌ Collect fitted Plateau and kobs values

Ligand conc (nM)	Plateau (sensor units)	Kobs (min^{-1})
3,200	90.9	1.07
1,000	90.8	0.951
320	87.8	0.841
100	82.8	0.625
32	71.6	0.313
10	47.1	0.201
3.2	25.1	0.114

➍ Multiply Plateau and kobs values together

Ligand conc (nM)	Plateau × kobs (sensor units.min^{-1})
3,200	92.0
1,000	86.4
320	73.8
100	51.8
32	22.4
10	9.47
3.2	2.86

➎ Plot Plateau × kobs value vs ligand conc, & fit to sigmoid curve equation

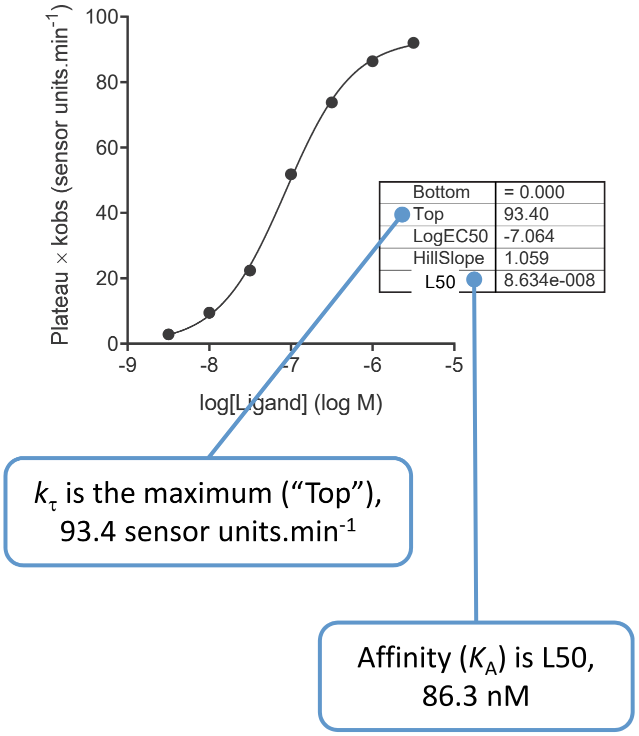

Supplementary Figure S6. Quantifying k_τ maximally-stimulating concentration method

1 Measure arrestin recruitment over time at a maximally-effective concentration

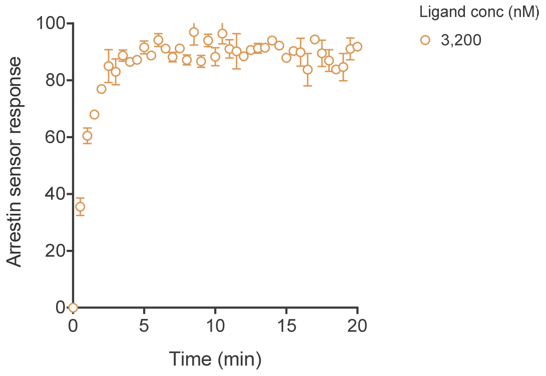

2 Fit data to association exponential equation

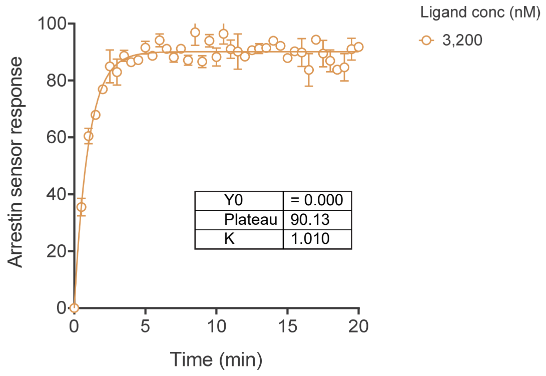

3 Collect fitted Plateau and kobs value and multiply them together. This is the k_τ value.

k_τ = Plateau $\times$ kobs = 90.1 $\times$ 1.01 = 91.0 sensor units.min^{-1}

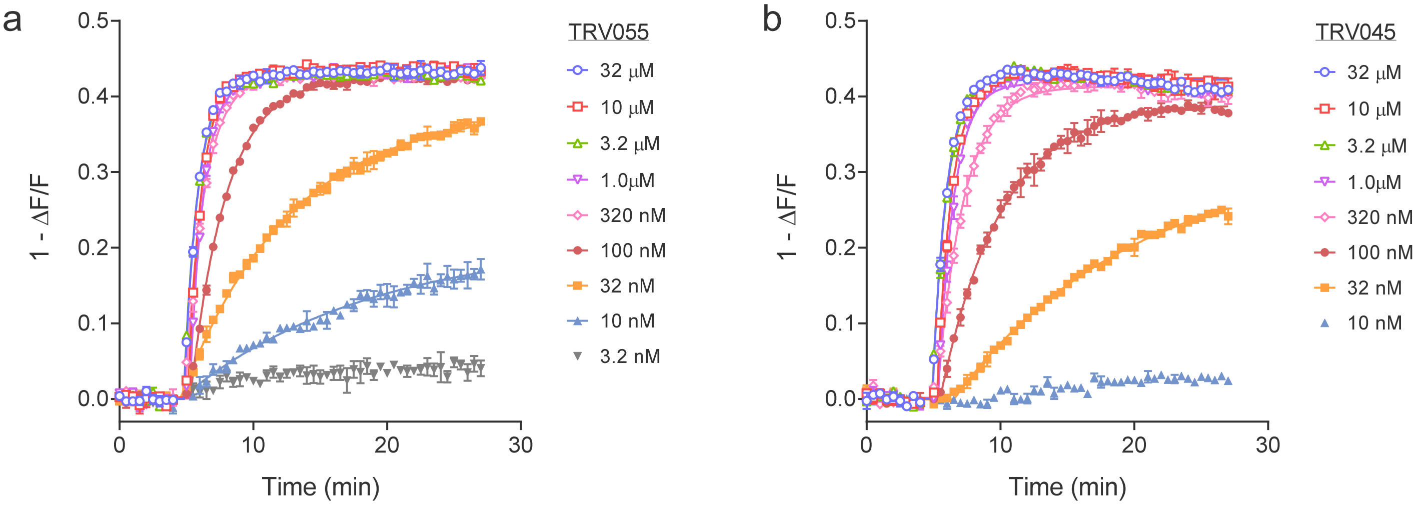

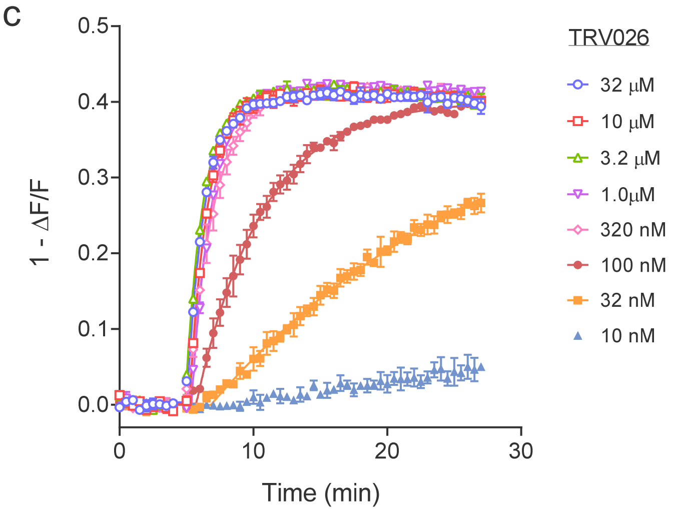

Supplementary Figure S7. Time course concentration response of arrestin recruitment to the AT_1 angiotensin receptor stimulated by TRV055 (a), TRV045 (b) and TRV026 (c). Curves are the fits to the association exponential equation:

$$y = \text{Plateau} \times \left(1 - e^{-k_{obs} . t}\right)$$

From the fitted value of k_{obs} and Plateau k_τ can be calculated as described in Fig. 2. Data were normalized as described in Fig. 2. Data are from the Biotek Synergy Mx plate reader.
